# Distress, Risk Factors, and Adaptive Strategies of Middle Nurse Managers in Italy

**DOI:** 10.3390/healthcare14142233

**Published:** 2026-07-22

**Authors:** Ilenia Piras, Igor Portoghese, Marco Vivolo, Giulia Adamo, Maura Galletta

**Affiliations:** 1 Clinical Trials Sector, General Affairs, ASL Cagliari, 09126 Cagliari, Italy; ilenia_78it@yahoo.it; 2 Department of Medical Sciences and Public Health, University of Cagliari, 09100 Cagliari, Italy; igor.portoghese@unica.it; 3 Emergency Department, SS. Trinità Hospital, ASL Cagliari, 09121 Cagliari, Italy; ma90@live.it; 4 “D. Casula” Hospital Department, AOU Cagliari, 09042 Cagliari, Italy; giuli.aa@hotmail.it

**Keywords:** well-being, middle nurse manager, hospital, stressor, working group, coping strategies, Italy

## Abstract

**Background/Objectives:** The role of middle nurse managers is characterized by high levels of responsibility and complex relationships, which can generate significant stress and put their mental health at risk. A mixed-methods study was carried out to measure distress and work-related factors among Italian middle nurse managers and to explore their experiences in order to identify critical issues and possible actions for improvement. **Methods:** The study was conducted between October 2019 and January 2020. **Results:** In the quantitative phase, 65 middle nurse managers completed validated questionnaires on distress, perceived health, working environment, job satisfaction, and emotional workload. Although most participants reported feeling healthy, nearly 30% experienced distress ranging from mild to severe. These findings informed the qualitative phase, in which 10 middle nurse managers were interviewed to further explore the lived experiences. Qualitative analysis identified four main themes: (1) sources of stress at work; (2) the importance of personal and organizational support for middle nurse managers; (3) copingstrategies; (4) actions for change to improve work activities. All themes were further divided into sub-themes. Middle nurse managers report experiencing difficulties in their work, adopting strategies to adapt to everyday challenges, and expressing a need for support to improve the management of their work teams. **Conclusions:** The findings suggest that, despite generally positive well-being indicators, a substantial subgroup of middle nurse managers experience distress related to organizational constraints. Practical implications include strengthening organizational support, reducing excessive workload, and addressing staffing issues to better sustain middle nurse managers’ well-being.

## 1. Introduction

Promoting mental health at all organizational levels of healthcare companies is a challenge. The organizational pyramid of healthcare companies includes different roles and skills: at the top is strategic management, then middle management, and finally the operational core with the various healthcare services and healthcare and administrative professionals. Middle nurse managers are in a particularly delicate position as they act as a link and integrator between strategic management and the operational level [1]. Performing a managerial role such as theirs can pose challenges for mental well-being, linked precisely to the type of role they hold. Middle nurse managers have a complex role due to their numerous responsibilities in terms of managing resources, assigning staff, and achieving organizational results [2]. The literature indicates that these professionals experience stress from managerial workloads, a lack of support and professional autonomy, repercussions of work on their personal lives, and high levels of self-perceived distress [3,4]. Among the most frequently reported critical issues by nurse managers are work overload, lack of time to perform tasks, and problems in managing relationships with the team and stakeholders [5,6]. They face high levels of work stress on a daily basis, with a significant impact on their psychological well-being, the onset of burnout, and their work performance [7,8,9]. The risk of negative outcomes for their mental health is greatly increased by the stress experienced at work, which negatively affects both their personal lives and relationships outside the workplace [7]. In addition, staff shortages in the services where they work also increase the risk of mental health problems, increasing the likelihood of burnout [10]. Personal resources, such as self-efficacy and psychological condition, positively influence work life quality and mental well-being, and increase workers’ ability to implement positive coping strategies [3,4,6]. According to the Job Demands-Resources (JD-R) model [11], well-being at work is shaped by the balance between job demands and the availability of organizational and personal resources. In line with this framework, nurse managers’ well-being is influenced not only by demands inherent in their role, but also by the availability of organizational resources, leadership climate, and opportunities for professional support [4,12]. When workload is excessive and role expectations are not matched by adequate resources, nurse managers may experience elevated psychological distress, emotional burden, and work-related strain [13,14,15]. Conversely, evidence suggests that self-efficacy, coping resources, and supportive work environments are associated with better mental well-being, stronger resilience, and greater capacity to sustain leadership responsibilities over time [6,16,17]. Moreover, providing adequate organizational support can improve leadership and resilience for middle nurse managers and help protect them from burnout [16]. A supportive approach enhances skills and overall well-being of the professionals, while also promoting their retention and organizational well-being in multidisciplinary work groups [18,19].

Although existing research addressed various occupational aspects of the nursing managerial role, evidence specifically focused on middle nurse managers is still limited. This gap is especially relevant in the context of Italian hospitals, where organizational structure, staffing models, and managerial responsibilities may influence the experiences of nursing leadership in ways that might not be fully captured by the broader international literature, therefore requiring a specific investigation. For this reason, a mixed-methods design is particularly appropriate. A quantitative phase would allow for assessing levels of distress and perceptions of the working environment, while the qualitative phase would makes it possible to deepen findings by exploring middle nurse managers’ lived experiences, critical issues, and suggestions for improvement. The integration of both approaches is therefore essential to obtain a more comprehensive understanding of the phenomenon under study.

## 2. Study Aim

An exploratory study design with mixed-methods was used. The aim of this study was to explore organizational well-being of middle nurse managers who manage professional teams in hospital settings. More specifically, using a mixed-methods approach, we first assessed the levels of distress and perception of the working environment (e.g., support, working relationships, job demands, workload). Moreover, we investigated possible significant differences in the studied variables with regard to working context and intention to move from the ward. Second, middle nurse managers’ stressful working experiences and coping strategies were explored to identify any critical issues and proposals for improving well-being in the workplace.

## 3. Materials and Methods

A mixed-methods approach was adopted, starting with a quantitative phase that used validated scales measuring distress, perceived health, working environment, and job satisfaction. Based on the findings of the quantitative analysis, a qualitative approach was used with semi-structured interviews with a subgroup of middle nurse managers. The study was conducted in three Italian hospitals belonging to two healthcare organizations in an Italian region.

### 3.1. Participants

The study participants were middle nurse managers who coordinate clinical care activities in hospital settings (e.g., medical wards, surgical wards, and services) and organize the work activities of multidisciplinary professional teams (e.g., nurses, social health workers, other health professionals). The middle nurse managers involved in this study have direct responsibility for the organization of care wards and include roles such as head nurse and ward nurse. The hospital settings where the middle nurse managers involved in the study work are:

- Medical area: Gastroenterology, Infectious Diseases, Internal Medicine, Cardiology, Spinal Unit;
- Surgical area: General Surgery, Otolaryngology, Maxillofacial Surgery, Obstetrics and Gynecology, Orthopedics, Pulmonology, Urology;
- Operating rooms or multidisciplinary surgical unit;
- Critical care: Resuscitation, Coronary Intensive Care Unit, Emergency room;
- Services: Radiology, Laboratory, Nephrology and Dialysis.

The inclusion criteria for participants were (a) working as a middle nurse manager in a hospital at the time of the study; (b) having worked as a middle nurse manager for at least 6 months; (c) voluntarily participating in the study.

### 3.2. Procedures

Once authorization had been obtained from hospital management, 72 eligible middle nurse managers at the participating hospitals were identified. Two members of the research team contacted them by phone to invite them to participate in the study. During the call, the study’s aim and data collection methods were explained. Interested middle nurse managers were then invited to an in-person meeting, where they provided informed consent and completed an anonymous paper questionnaire The questionnaires were administered in October 2019, and 65 middle nurse managers returned them completed. After completion, each participant placed the questionnaire in a sealed envelope provided by the research team to ensure privacy. The research team collected the sealed envelopes a few hours or days later. At the time of the data collection, all 65 middle nurse managers were invited to participate in a semi-structured interview on organizational well-being. Among them, 19 people agreed to be interviewed and were subsequently contacted to arrange a convenient time and place, ensuring confidentiality and anonymity. The interviews were conducted in January 2020 in person or by telephone, depending on the participants’ preferences. An interview guide was developed based on the preliminary results from the questionnaires in order to explore certain aspects or critical issues that had emerged. The guide was used flexibly to allow the interviewee to share their experiences without restrictions. Prompting questions such as “Can you explain that better?”, “Why?”, and “Can you give an example?” were asked throughout the interview to allow the interviewee to elaborate and detail their experiences. The interviews were recorded with informed consent and were conducted until saturation was reached. Saturation was considered reached when no new meaningful codes or thematic categories emerged from the final interviews, indicating that the collected data were sufficient to fully capture the phenomenon under study. All data (quantitative and qualitative) were processed in compliance with privacy regulations (Italian Decree-Law 196/2003).

### 3.3. Instruments

#### 3.3.1. Questionnaire

The quantitative survey was conducted using a structured questionnaire composed of validated scales. The questionnaire included a demographic section (gender, age, hospital setting, years of experience in the setting where they work, intention to request a transfer to another facility) and a section with measurement scales related to well-being at work. Specifically, the questionnaire included the following scales:

Distress. To measure psychological distress, the Kessler Psychological Distress Scale (K10) of 10 items was used [20]. The Likert scale ranged from 1 = None of the time to 5 = All of the time. Scores ranged from 10 to 50. Middle nurse managers who score under 20 are likely to be well, those who score 20–24 are likely to have a mild mental disorder, those who score 25–29 are likely to have moderate mental disorder, and those who score 30 and over are likely to have a severe mental disorder.

HSE. To measure stress-risk factors, the Italian version (ISPESL-HSE) of the HSE questionnaire by Edwards et al. was used [21]. The dimensions investigated in the study are demand (9 items), job control/autonomy (5 items), role clarity (5 items), relationships with colleagues/supervisors (6 items), and support from colleagues/supervisors (7 items). The Likert scale ranged from 1 (always) to 5 (never).

Perceived Health. The WHO-5 scale of 5 items was used to measure perceived mental well-being [22]. The Likert scale ranged from 0 (never) to 4 (daily).

Job Satisfaction. Three items by Cammann, Fichman, Jenkins, & Klesh [23] were used to measure job satisfaction. The Likert scale ranged from 1 (completely disagree) to 5 (completely agree).

Emotional Workload. The 5-item sub-dimension of the Areas of Worklife Scale—AWS by Leiter and Maslach was used [24]. Items were rated using a 5-point scale ranging from 1 (strongly disagree) to 5 (totally agree).

#### 3.3.2. Interview

The qualitative survey was conducted using an ad hoc interview guide. The interview guide was developed after the preliminary analysis of the quantitative data, with the aim of exploring in greater depth the main critical aspects emerged from the questionnaire. In particular, the guide focused to explore middle nurse managers’ work experiences, their perceived role, their relationships with staff and supervisors, and their perceptions of the working environment. The interview questions were therefore shaped to clarify and contextualize the quantitative findings by eliciting participants’ experiences, perceptions, and explanations regarding the critical issues identified in the first phase of the study. The guide was used flexibly to allow participants to further elaborate on relevant topics and introduce additional issues they considered important. The questions contained in the interview guide are as follows:

1. What are the main critical issues you deal with in your work as a middle nurse manager?
2. How do you deal with daily work problems and difficulties?
3. How do you experience your role as a middle nurse manager?
4. How would you describe the interaction between you as a middle nurse manager and your company or your supervisors?
5. How would you describe the interaction between you as a middle nurse manager and the staff where you work?
6. What do you think would enable you and your staff to work optimally?

### 3.4. Analysis

Quantitative data were analyzed using SPSS version 20.0 software. To determine whether the sample size of 65 participants was adequate, a post hoc power analysis was carried out using G*Power 3.1 [25], for a two-tailed one-sample *t*-test, assuming an effect size of 0.5 and α error probability of 0.05 [26]. The quantitative study was conducted for descriptive and comparative purposes. To test reliability of the scales, we applied Cronbach’s Alpha (α). Simple frequency and percentage analyses and descriptive analyses were carried out to analyze the demographic and descriptive characteristics of the sample. Non-parametric tests for non-adjusted comparisons between groups was used to assess differences in the analyzed variables. Specifically, the Kruskal–Wallis with post hoc comparisons (*p* < 0.05) was applied for length of service in the ward, working area, and request for moving to another ward. Qualitative data analysis was performed using Colaizzi’s method [27]. Interviews were transcribed faithfully and verbatim, including not only verbal content but also significant paraverbal elements such as pauses, voice inflections, laughter or sighs, and annotations on nonverbal context (e.g., gestures, facial expressions) useful for understanding meaning. The analysis was conducted by two members of the research team with experience in qualitative analysis. Before the formal coding phase, they discussed the analytical framework and the coding procedures to align their approach and ensure consistency in interpretation. To enhance rigor and trustworthiness, each researcher reviewed the transcripts independently to become familiar with the content and identify initial codes. This first coding step allowed them to extract meaningful statements and key concepts from each transcript, thus generating a preliminary list of thematic categories and subcategories. In the next step, the researchers conducted a series of collaborative discussions to compare the initial codes and categories. Through a process of in-depth code analysis, related codes were grouped together and emerging thematic dimensions were identified. Although a formal intercoder agreement coefficient was not calculated, discrepancies were discussed until agreement was reached. This process resulted in lists of central themes identified by the researchers, which were then jointly discussed and refined to reach consensus on the main themes. To triangulate the data, the researchers compared and validated emerging themes not only across interviews, but also taking into account their different perspectives and professional experiences. This process strengthened the validity and reliability of the results, ensuring a richer and more comprehensive understanding of the phenomena studied. Following joint discussion and further selective coding, the final central themes representing the core of the analysis were finalized. Although the interviews were conducted in Italian, after analyzing and consolidating the themes from the transcripts, relevant quotations from middle nurse managers were translated into English, maintaining maximum fidelity to the original meaning.

### 3.5. Ethical Considerations

Participants were informed about the purpose of the study and participated voluntarily and anonymously in accordance with Italian privacy law (Legislative Decree No. 196/2003, Legislative Decree No. 101/2018). Middle nurse managers provided informed consent prior to the interview and were guaranteed answers to any questions they asked the researchers. The interviews were recorded with the consent of the participants, and a unique sequential identification code was assigned to ensure anonymity (ID). The middle nurse managers had the opportunity to raise any concerns regarding anonymity and data collection. They were properly reassured and supported by the research team, who provided all the necessary information about the procedures and ensured that participants could speak freely without negative consequences. It was also clarified that the use of anonymous questionnaires, non-identifiable codes, and confidential interview settings further minimized any concerns related to confidentiality when discussing sensitive organizational topics. This research complies with the Declaration of Helsinki and the General Data Protection Regulation (EU) 2016/679 (GDPR). Participants were informed that they had the right to withdraw from the study at any time without negative consequences.

### 3.6. Reporting Guidelines

This study was reported in accordance with reporting guidelines appropriate to the study design. The quantitative observational phase was reported with reference to the STROBE recommendations, the qualitative interview phase was described in line with the COREQ checklist, and the overall mixed-methods design was presented considering the GRAMMS framework. These reporting guidelines were used to improve methodological transparency, completeness of reporting, and reproducibility of the study procedures and findings.

## 4. Results

### 4.1. Quantitative Analysis Results

#### 4.1.1. Statistical Power

The power analysis showed that, with a sample size of 65 individuals, the statistical power achieved was 97.8%, thus suggesting that the sample size was adequate for the study.

#### 4.1.2. Demographics

Among the 72 eligible middle nurse managers, 65 were included in the study (90.3%) response rate); the majority were female (*n* = 52, 80%), with males comprising 20% (*n* = 13). Regarding working area, the largest proportion of participants worked in the medical area (*n* = 18, 27.7%), followed by the services (*n* = 16, 24.6%) and critical area (*n* = 15, 23.1%). Smaller percentages were observed in operating rooms (*n* = 10, 15.4%) and the surgical area (*n* = 6, 9.2%). Ward tenure varied among participants. The largest group of middle nurse managers had over 10 years of experience (*n* = 38, 58.5%), followed by the participants with 4–10 years in the service (*n* = 18, 27.7%). The lowest percentages were among participants with 1–3 years (*n* = 7, 10.8%) or less than 1 year (*n* = 2, 3.1%) of experience in the service. Most of the sample did not request ward moving (*n* = 55, 84.6%), and 15.4% of middle nurse managers (*n* = 10) did so (Table 1).

#### 4.1.3. Distress Levels

The sample shows a median distress score of 16 (range 10–37), indicating good health status among middle nurse managers. Specifically, while the majority of the sample, about 71% (*n* = 46), report themselves as healthy with a median score of 14 (range 10–19), a considerable portion (*n* = 29%) face distress (X^2^ = 73.2, df = 3, *p* < 0.001). Specifically, 12% (*n* = 8) have severe distress (median score 32.5, range 30–37), 11% (*n* = 7) moderate distress (median score 26, range 25–29), and 6% (*n* = 4) mild distress (median score 20.5, range 20–24). (Table 2). Comparing distress levels between middle nurse managers working in critical areas (*n* = 19) versus noncritical areas (*n* = 46), both subgroups showed the same median distress score of 16 (range 10–34 for critical area and 10–37 for noncritical area). Table 2 details the frequencies and percentages of distress for each working area. The results indicate that there are no significant differences in distress levels between middle nurse managers in critical areas and those in non-critical areas (X^2^ = 0.722, df = 3, *p* = 0.868).

#### 4.1.4. Descriptive Characteristics

Table 3 presents descriptive statistics (the median values, with minimum and maximum) and Cronbach’s Alpha (α) for all of the study variables. Participants report median values of 1.00 for distress and role clarity, suggesting that studied middle nurse managers tended to have very low levels of distress and perceived high role clarity. The median values of 3.00 for demand, relationships with colleagues/supervisors, and support from colleagues/supervisors indicate that participants perceive they have moderate levels of these variables, where they sometimes feel supported and feel they have good quality relationships, and sometimes feel they have high demands. Middle nurse managers report a median of 2.00 for job control, suggesting that they perceive a relatively high level of autonomy and feel they often have control over their work. Middle nurse managers report a median job satisfaction score of 4.00, indicating that they are highly satisfied with their job. Although there is some variability in the responses (with a range of 1.00 to 5.00), the overall trend is towards higher levels of satisfaction. Perceived health has a median value of 4.50, suggesting that participants tend to perceive their health on a regular basis, with some middle nurse managers perceiving it more frequently. Finally, emotional workload has a median of 3.00, with a range of 1.50 to 4.00. This shows that emotional workload is perceived at a moderately high level in the study sample.

The Kruskal–Wallis test for subgroup comparisons found no significant differences between groups according to working area and length of service in the ward for any variable analyzed (*p* > 0.05). Comparing middle nurse managers who requested to move to another ward (*n* = 10, 15%) versus those who did not (*n* = 55, 85%), significant differences emerged for most of the studied variables (Table 4). Specifically, those who requested a ward transfer reported higher levels of distress (median = 3.00) in contrast to those who did not request a transfer, who showed lower median distress (median = 1.00). This subjective experience of discomfort was further underscored by significant differences in perceived health, with the group desiring a move reporting poorer health (median = 3.00) compared to a higher median for the other group (median = 5.00). In terms of perceived working environment, the group requesting a ward change indicated lower job control (median = 3.00 vs. 2.00 in the other group) and lower job satisfaction (median = 2.00 vs. 4.00). Conversely, no statistically significant differences were found for demands (χ^2^ = 1.14, *p* = 0.28), role clarity (χ^2^ = 3.39, *p* = 0.07), relationship with colleagues/supervisors (χ^2^ = 0.01, *p* = 0.91), support from colleagues/supervisors (χ^2^ = 3.06, *p* = 0.08), and emotional workload (χ^2^ = 3.44, *p* = 0.06).

### 4.2. Qualitative Analysis Results

Of the 19 middle nurse managers who volunteered for the interview, 10 were ultimately interviewed. The remaining nine were not included because data saturation was considered to have been reached after the tenth interview, as no new relevant codes or themes emerged. The interviewees were mainly female (*n* = 7) and the average age ranged from 30 to 60 years. All hospital work settings were adequately represented, with most participants working in the medical area (*n* = 3), the surgical area or operating rooms (*n* = 3), and the critical care area (*n* = 2). Most had more than 10 years of experience as middle nurse managers (*n* = 5, 50%), followed by participants with 4–10 years of experience (*n* = 4, 40%) (Table 5).

#### Main Themes and Sub-Themes

Interviews with middle nurse managers identified four main themes related to their experience of organizational well-being at work. These themes include (1) sources of stress at work; (2) the importance of personal and organizational support for middle nurse managers; (3) coping strategies; and (4) actions for change to improve work activities. All themes were further divided into three sub-themes, with the exception of theme 4, which was divided into two sub-themes. A summary of the themes and sub-themes is presented in Table 6.

Theme 1. Sources of stress at work

This theme highlights the factors that contribute to increased stress among middle nurse managers. They feel that they are often overworked due to too many tasks to perform and that they have to deal with critical situations on a daily basis due to organizational shortcomings that are beyond their control (e.g., staff shortages). This situation also affects the good working relationships within the professional team they manage and leads them to have to deal with the resulting conflicts.

Sub-theme 1.1. Work overload due to too many tasks to perform

In the following interviews, middle nurse managers described the factors that contribute to their overload during their shifts, expressing discomfort and discouragement at not having enough time to do everything that needs to be done.

*“… There are high expectations from the staff, but there is not enough time during the day [work shift] to organize meetings, respond to the various needs of the staff, talk to patients, and listen to complaints from families” (ID3).*

*“Our job [as middle nurse managers] is to manage, plan, think strategically (…) but, day after day, I realize that I never get to do what I had in mind. I am forced to constantly interrupt my work, I have to meet deadlines, manage consultations [with hospitalized patients], make arrangements [for the various activities of the ward], order drugs and medical devices, order meals… I am never at ease” (ID7).*

Sub-theme 1.2. The serious problem of staff shortages

The interviewees are aware that they cannot solve the major problem of staff shortages in the teams on their own. They describe what they are trying to do to improve the situation, but also the consequences that this problem has on daily care.

*“This is the most uncomfortable part of my job, because I can’t make human resources appear. I keep fighting with the healthcare manager and the nursing office to get more staff, to make the nurses’ work easier and ensure patient well-being, but I always get negative responses” (ID8).*

*“There are few nurses and they ask me for help. I see them, they are tired, they don’t enjoy their work, and this is particularly evident when caring for patients and in conflicts between staff members themselves” (ID10).*

Sub-theme 1.3. Team relationships and conflict management

The balance within the work group is undermined both by resistance to change and by negative behavior on the part of some professionals, which creates an unpleasant atmosphere and triggers conflicts.

*“Some [staff members] are uncooperative and manipulative, trying to involve and drag other colleagues into negative behavior; this can also have a harmful effect on the group” (ID1).*

*“I realize that older staff members are more resistant to change and hinder the innovative proposals of their younger colleagues” (ID5).*

Theme 2. The importance of personal and organizational support for middle nurse managers

Middle managers express the need to feel more involved in the decisions of the company where they work. Respondents complain that they have to deal with tasks that are not part of the remit of middle nurse managers and would like to be helped to carry out certain activities during their work. In addition, they report needing emotional support and being aware that their well-being has positive or negative effects on the staff they manage.

Sub-theme 2.1. The need for emotional support and feeling involved in the organization

This sub-theme indicates middle nurse managers’ feelings about their need to receive emotional support from fellow middle managers. In addition, they would like to be given greater consideration and involvement by company executives in business decisions that affect them.

*“It would be useful to be more involved in company strategies. In general, I don’t feel supported (…) then sometimes decisions are made directly by upper management and you just have to adapt” (ID2).*

*“I would feel much more at ease if I had the opportunity to share all my concerns with other coordinators after work and receive adequate support from managers” (ID10).*

Sub-theme 2.2. Respect for the skills of middle nurse managers and the need for support

The skills of middle nurse managers are not respected and they find themselves engaged in activities that are not their own, resulting in work overload. If other figures took care of these activities or if some staff members helped them to carry them out, they would be able to perform their own duties better.

*“I think we perform many functions that we shouldn’t be performing. We are overloaded. Instead, it would be enough to share some tasks with staff members, reduce bureaucracy, and have greater collaboration between services and management” (ID1).*

*“Each of us [middle nurse managers] would need someone to help us with some of the tasks so that we could focus on those activities that really concern our role and not all those that we perform due to a lack of other human resources” (ID6).*

Sub-theme 2.3. Effects of personal well-being on staff

Respondents say that the team is influenced by the mood of their middle manager. Therefore, they recognize that it is important to remain calm and composed in order to convey harmony and security within the work group.

*“… as a middle manager, you can make the [work] environment positive or negative; the group is influenced by you, so you have to stay calm and know how to create harmony in the group” (ID4).*

*“When I am calm, I can convey confidence to those who work with me. If I am nervous, I convey nervousness to everyone; I think they [my colleagues] are affected by my mood” (ID7).*

Theme 3. Coping strategies

There are several strategies that middle nurse managers use to adapt to work situations (e.g., establishing rules and priorities for carrying out activities). Among the personal strategies, they report that isolating themselves can allow them to find solutions to problems that arise. Being flexible and knowing how to adapt to what happens on a daily basis is another strategy they use. In addition, they use the active involvement of other professionals in the situations they face as a strategy.

Sub-theme 3.1. Personal strategies used at work

In the following interviews, middle nurse managers described the strategies they use while working to best manage various situations. They report that it is important to take the time to reflect if the situation is not urgent, establish clear rules with the team, and deal with priority problems first.

*“I take my time, unless an immediate response is needed. I usually set parameters with the staff (my staff calls it ‘democratic dictatorship’) and if the group does not decide, I will, but everyone needs to take responsibility for their own choices” (ID6).*

*“I try to solve problems one by one, perhaps adopting other strategies, focusing and leaving out minor issues” (ID9).*

Sub-theme 3.2. Involvement of the work group

This sub-theme shows that middle nurse managers use work group involvement as an effective strategy. This allows them to plan various activities together with professionals and also to resolve any problems that may arise during work shifts together. In addition, it is also perceived as a form of support and help for the middle nurse manager and members of their team.

*“We all meet at 7 a.m. and while we have coffee, we talk and plan the day, so where possible, we will solve any problems that arise during the shift” (ID2).*

*“Working as a team and communicating with colleagues, involving them in the situation we are facing, is often more effective in addressing issues and helping us middle managers deal with stressful situations. When I talk to someone about it, I feel better, it helps me. We support each other, and I think that’s important. That’s what really makes the difference. That’s what helps” (ID3).*

Sub-theme 3.3. Isolation and ability to detach

This sub-theme highlights that middle nurse managers sometimes isolate themselves from the rest of the team and from work problems. They report that they detach themselves and engage in other activities that help them solve these problems more effectively. In addition, once they have left their shift, they try to distance themselves mentally from work-related issues.

*“I isolate myself for a moment and try to detach myself from the problems. When I leave, I try not to think about them anymore, devoting myself to other activities that give me satisfaction and take me away mentally and physically from work” (ID5).*

*“I have a coffee, take a break, and go for a walk outside the office. After that, I usually tidy up, for example, the medicine storage room, and in the meantime, I think about how I can solve the problem” (ID8).*

Theme 4. Actions for change to improve work activities

This theme shows that, according to middle nurse managers, their work could be improved by having more appropriate work tools (e.g., information technology) at their disposal. In their opinion, having professionals with greater skills would trigger positive changes such as safer patient care. They also believe that companies should motivate professionals and retain them in order to avoid high turnover rates.

Sub-theme 4.1. Motivating professionals and retaining them in the company

Middle nurse managers believe it is important to take action to motivate professionals and improve their working conditions. Companies should invest more in the individual professionals who work within the teams and retain them to avoid high turnover rates, which then have an impact on the functioning of the healthcare company and the care provided to patients.

*“The work of a nurse and the responsibilities it entails make it an increasingly unattractive profession. If nurses are not adequately motivated and find themselves working in unsafe conditions, the situation will become increasingly difficult to deal with. We need new, young people who are eager to work and improve” (ID9).*

*“Training in stress management and conflict management is not enough to deal with the problems of the work group. We need to recruit staff and, once trained, hold on to them and not let them slip away” (ID10).*

Sub-theme 4.2. Improving work tools, professional skills, and care safety

There are several actions that could help improve the work of professionals and that middle nurse managers suggest. They report the need for appropriate and up-to-date IT tools to be able to work. In addition, they would like to see investment in improving the skills of professionals, including the introduction of organizational models that contribute to making patient care safer.

*“I would like to see the introduction of a primary nursing role to coordinate a group of nurses and liaise with me on the most important issues; this person would be a point of reference, but would need to have a certain amount of authority and expertise in this area” (ID2).*

*“I would like to see an appropriate and faster IT system so that we can work better, for example, to arrange work shifts, order medications, and manage nursing records. Unfortunately, most of the time, the IT system is very slow, and I have to waste a lot of time at the computer!” (ID4).*

### 4.3. Integration of Quantitative and Qualitative Findings

The results of the comparisons between middle nurse managers who applied for a transfer vs. those who did not were aligned with the qualitative themes. The joint display shown in Table 7 provides an integrated interpretation of the data, underscoring the correspondence between the narratives of middle nurse managers and the observed quantitative patterns, particularly with regard to distress, perceived health, control at work, and job satisfaction. Specifically, the integrated results show that middle nurse managers who expressed a desire to change departments report higher levels of distress, poorer health, less control over their work, and lower job satisfaction compared to those who did not express such an intention. The qualitative data help interpret these findings, indicating that the main sources of distress are work overload, frequent interruptions, staff shortages, the burden of tasks inconsistent with their role, and the perception of a lack of recognition from the organization. Furthermore, the interviews show that participants adopt coping strategies based on flexibility, prioritization, discussion with colleagues, and temporary emotional detachment—elements that help them maintain a functional balance despite the difficulties. Finally, the qualitative data highlight a call for organizational change, with particular emphasis on greater support, more adequate work tools, involvement in decision-making, and recognition of professional skills.

## 5. Discussion

The results of this study show an overview of well-being perceptions of middle nurse managers, offering useful information about the characteristic of their work environment. The quantitative results indicate that, although most middle nurse managers reported feeling healthy, nearly 30% experienced mild to severe distress. This subgroup deserves particular attention, as it represents a significant proportion of professionals who may be exposed to a considerable clinical and organizational workload. The presence of distress—even if mild to severe—suggests that a substantial minority of the sample may require targeted organizational support and timely preventive measures [28].

A significant finding from the descriptive analysis concerns distress and role clarity. Middle nurse managers reported low levels of distress and high role clarity, thus suggesting that many participants would have a clear understanding of their responsibilities and generally perceived their role as well defined. Job demands, relationships with colleagues and supervisors, and organizational support were reported at moderate levels, thus indicating that these dimensions would be present but not consistently experienced as optimal. Participants also perceived a relatively high level of control over their work, thus suggesting a fair degree of autonomy in organizing daily activities. Job satisfaction was high in the sample, and this positive result, together with generally good perceived health, would contribute to a favorable overview of well-being [29]. However, emotional workload was reported as moderately high and this may indicate that emotional strain remains a relevant feature of the role, even when general satisfaction appears high.

An interesting result of the study concerns middle nurse managers who requested a transfer to another ward. Compared with those who did not express this intention, they reported higher distress, poorer perceived health, lower job control, and lower job satisfaction. This pattern would suggest that the desire to transfer may be a marker of broader occupational strain rather than a simple preference for mobility. In other words, the request for transfer appears to reflect a critical appraisal of the current work context, in which distress and dissatisfaction coexist with reduced control over work activities. These findings would support the literature showing that higher workload-related stress is associated with lower job satisfaction and greater intention to leave [30,31]. Overall, the results would suggest that retention issues among middle nurse managers may likely be linked to organizational conditions in which their role is enacted. In this sense, it may be [18].

Qualitative results help to deep and explain quantitative data. In fact, the interviewed middle nurse managers highlighted the persistent staff shortage, the excessive workload, frequent interruptions, and tasks outside their formal role as the main sources of stress. These factors would align with the lower job control and job satisfaction reported among those who requested a ward transfer. In particular, carrying out tasks outside their role would reduce the time available for main managerial responsibilities and increase the perception that the role is overloaded and poorly protected.

Regarding sources of stress, the interviewees talked about a mismatch between the amount of work request and the time available to complete it. This imbalance can result in discouragement and a sense of inadequacy in managing one’s responsibilities [6,32]. The literature shows that unreasonable workloads increase the risk of job stress and burnout among nursing leaders [13,33]. The interviewees also highlighted frequent interruption of ongoing activities to respond to urgent and unexpected requests, which would fragment work and make planning of care more difficult [31]. While such unpredictability is typical in healthcare settings, it may become particularly burdensome when it is not supported by adequate staff and organizational resources [34]. However, the literature would suggest that greater control over time and task distribution would likely help nurse managers perform their leadership roles more effectively [35]. A relevant issue emerged from participants’ narratives concerns activities that fall outside their formal role. This aspect could better explain the lower scores of job control reported by the subgroup requesting transfer. When middle nurse managers must continuously manage responsibilities that do not belong to their formal remit, their role would become blurred and more difficult to sustain [32]. From an organizational standpoint, this would suggest the need to analyze workloads more carefully and adopt work arrangements that better respect role boundaries and responsibilities. Delegation, more effective task distribution, or care models that reduce unnecessary organizational burden may help free time for core managerial functions and improve the overall balance of work within the team [34].

Staff shortages emerged from the narratives as another major source of stress. Participants described a severe lack of staff and emphasized that this was a structural constraint rather than an individual failure. This aspect is important because it would frame distress as a response to organizational conditions rather than a personal weakness. The literature supports that nurse managers devote substantial time to managing staffing gaps and that this burden can generate stress and negative perceptions of human resource adequacy [31]. Staff shortages also would increase burnout risk and other mental health problems among middle nurse managers [10]. In the present study, these shortages may be tied to the distress pattern and low job satisfaction observed among middle nurse managers requested transfer, thus suggesting that staffing adequacy could be a central determinant of occupational well-being.

The interviews also showed that staff shortage make team management more difficult, especially when the work environment becomes tense or resistant to change. Participants reported interpersonal conflict and counterproductive behaviors within teams, which further complicated coordination and undermined group functioning. These issues may partly explain the low job satisfaction scores reported by the group of middle nurse managers who requested a transfer. The literature emphasizes the importance of strengthening communication and professional exchange between nursing managers and staff, as closer interaction can improve collaboration and mutual understanding [36]. Participatory leadership styles can be particularly useful in this regard, as they help address staff needs and promote job satisfaction [9].

Another interesting aspect emerged from the interviews concerns the link between middle nurse managers’ well-being and team functioning. Participants perceived their psychological and physical well-being as a prerequisite for maintaining positive relationships and effective leadership. This would suggest that their ability to lead team constructively depends on their own emotional condition. In this sense, organizational well-being at the managerial level may have a ripple effect on the functioning of the entire team [37]. This issue becomes even more relevant in relation to senior management, as participants expressed the need for greater consideration, involvement in decision-making, and recognition of their role. These findings align with the moderate levels of perceived support reported by participants. A culture of regular and constructive feedback may help middle nurse managers feel valued and supported, acting as a protective factor of well-being [18,29].

The coping strategies described by the participants would highlight how they seek to address workplace pressures. Middle nurse managers reported using adaptive approaches (e.g., cognitive and behavioral flexibility, prioritization, temporary detachment from the group), which also emerged in previous studies as protective resources [9]. In other cases, participants engaged the team through shift briefings and shared planning to manage daily activities more effectively. This also reflects the value of social support from colleagues, which is recognized in the literature for mitigating work-related stress in nursing management [14].

Middle nurse managers moved forward proposals to improve their own working conditions and those of their staff. They emphasized the need for better tools—including information technology—more appropriate models for organizing care, and greater investment in professional development. This would support the view that distress is linked to organizational factors rather than purely individual ones. In fact, improving well-being at work requires a supportive environment, recognition of professional roles, and structured training opportunities [4,5,6,10]. In this regard, healthcare organizations should actively invest in retaining and supporting nursing leadership by creating work environments that are both stimulating and sustainable, as evidenced by the literature [18,19]. Overall, the results can be interpreted through the theoretical framework of the Job Demands-Resources model, suggesting that working well-being of middle nurse managers would reflect their perception of demands and resources present in the workplace. The integration of quantitative and qualitative data would suggest that distress may stem in particular from staff shortages, tasks outside one’s role, excessive workload, and limited support. Specifically, the group of middle nurse managers who requested a transfer likely experience these challenges more intensely, with possible consequences for their health and job satisfaction. In this regard, concrete management policies aimed at reducing avoidable workloads, clarifying role boundaries, strengthening staffing level, and increasing organizational support are necessary.

### Limitations, Strengths, and Practical Implications

This study has some limitations. First, although a good sample size of middle nurse managers was obtained (considering the existing numerical proportion in the hospitals studied of one middle nurse manager for each team managed, consisting of approximately 30–50 professionals), it may not be representative of the issue of well-being at work among middle nurse managers in hospitals in Italy. Although the statistical power analysis showed that the number of participants in the study was representative of the population of middle nurse managers for the studied hospitals, the generalizability of these findings at the national and international levels should be interpreted with caution. The transferability of the results can be considered in similar organizational contexts, characterized by comparable healthcare system structures, nursing hierarchies, and size of care teams. Therefore, further multicenter studies in national and international settings are recommended to confirm and expand upon our findings.

Secondly, given the nature of self-reported data, it is not possible to completely rule out some influence of social desirability on the participants’ responses. However, the anonymous collection of data may have reduced this risk. Moreover, the data were collected at the same time, which may have introduced shared variance related to the measurement method. In this sense, common method bias may represent a possible limitation but likely marginal, as the data were used for descriptive and comparative purposes rather than to examine complex associations between variables.

Thirdly, future studies should also involve middle nurse managers in local services in order to analyze potential differences in sources of stress and coping strategies. This would provide a clearer and more complete picture of the issue in the various care contexts in which middle managers work. Fourthly, although the mixed-methods approach adopted in this study provided in-depth insights into the sources of stress and coping strategies of middle nurse managers, further investigation is warranted. Specifically, future research should examine the organizational conditions within the hospitals studied, as well as the strategies implemented to effectively support middle nurse managers in their roles. Moreover, the quantitative findings should be interpreted in light of the study’s descriptive comparative design. Since no multivariate analyses were performed, it cannot be ruled out that the observed differences may be influenced by potential confounding factors, such as age, tenure, work unit, or other characteristics of the sample. However, the analysis is consistent with the study aim, which was to describe and compare the sub-groups in an exploratory manner, rather than to estimate associations between the variables.

Finally, the data were collected in the 2019–2020 period, so the interpretation of the results needs to take into account organizational and care changes in the current context. However, we believe that the results remain relevant because they describe structural dynamics of managerial work that are still pertinent. Despite these limitations, the mixed-methods approach used in this study may contribute to a more comprehensive understanding of stress experienced by middle nurse managers and their ability to effectively cope with challenging situations.

The findings of this study suggest several practical implications for supporting middle nurse managers in hospital settings. First, given the reported overload and role ambiguity, organizations should consider reviewing task distribution and clarifying responsibilities to better align managerial duties with available resources. Second, the narratives highlight the importance of relational and organizational support; therefore, strengthening communication with supervisors, promoting opportunities for peer discussion, and fostering a more supportive work climate may help reduce distress. Third, the study suggests that middle nurse managers benefit from being more involved in decision-making processes that affect their work, as this may improve their sense of recognition and control. Finally, the reported need for adequate tools, staff collaboration, and a more manageable workload indicates that attention to day-to-day organizational conditions is essential to support well-being and work effectiveness.

## 6. Conclusions

Although the results suggest a general level of well-being among middle nurse managers, a notable portion of them experience distress, ranging from mild to severe. The group requesting a transfer from their ward shows a more critical profile, with higher distress and lower job satisfaction. Integration of qualitative data indicates that these difficulties may linked to staff shortages, tasks outside their role, and insufficient organizational support. Overall, the results highlight the need to regularly monitor distress, reduce workload, and strengthen organizational support for middle nurse managers. Future studies should include larger, multicenter, and longitudinal samples.

## Figures and Tables

**Table 1:** Sample demographics.

Variable	Level	*n*	%
Sex	Female	52	80
	Male	13	20
Working area	Surgical area	6	9.2
	Operating rooms	10	15.4
	Critical area	15	23.1
	Services	16	24.6
	Medical area	18	27.7
Ward tenure	<1 year	2	3.1
	1–3 years	7	10.8
	4–10 years	18	27.7
	>10 years	38	58.5
Request for ward moving	Yes	10	15.4
	No	55	84.6

Notes. N = 65.

**Table 2:** Distress level frequencies compared for working areas.

	Working Area			
Distress Level	Critical *n* (%)	Noncritical *n* (%)	Total *n* (%)	
Healthy (score < 20)	13 (68.4)	33 (71.1)	46 (70.3)	19 (29.7)
Mild distress (score 20–24)	1 (5.3)	3 (6.7)	4 (6.3)	
Moderate distress (score 25–29)	3 (15.8)	4 (8.9)	7 (10.9)	
Severe distress (score ≥ 30)	2 (10.5)	6 (13.3)	8 (12.5)	
Total	19 (100)	46 (100)	65 (100)	

**Table 3:** Descriptive characteristics for the study variables and Cronbach’s Alpha for reliability of the scales.

Variable	Median	Lowest	Highest	α *
Distress	1.00	1.00	4.00	0.92
Demands	3.00	1.00	5.00	0.76
Job control	2.00	1.00	5.00	0.86
Role clarity	1.00	1.00	5.00	0.74
Relationships with colleagues/supervisors	3.00	1.50	5.00	0.67
Support from colleagues/supervisors	3.00	1.00	5.00	0.84
Job satisfaction	4.00	1.00	5.00	0.64
Perceived health	4.50	1.00	7.00	0.96
Emotional workload	2.50	1.00	4.00	0.66

Note. Distress: 1 = never, 5 = ever; demands, job control, role clarity, relationship, and support: 1 = ever, 5 = never; perceived health: 1 = never, 7 = ever; job satisfaction and emotional workload: 1 = completely disagree, 5 = completely agree. * Cronbach’s alpha values greater than or equal to 0.60 are considered acceptable.

**Table 4:** Kruskal–Wallis test for ward moving request.

Statistics	Request for Ward Moving	Distress	Demands	Job Control	Clarity of Roles	Relationship with Colleagues/Supervisors	Support from Colleagues/Supervisors	Perceived Health	Job Satisfaction	Emotional Workload
Median	Yes	3.00	2.50	3.00	2.00	3.00	3.00	3.00	2.00	3.00
	No	1.00	3.00	2.00	1.00	3.00	3.00	5.00	4.00	2.50
Lowest	Yes	1.00	1.00	2.00	1.00	1.50	2.00	1.00	1.00	2.00
	No	1.00	1.00	1.00	1.00	1.50	1.00	2.50	2.00	1.00
Highest	Yes	3.50	4.00	4.00	5.00	4.50	3.00	6.00	5.00	4.00
	No	4.00	5.00	4.00	4.00	5.00	5.00	7.00	5.00	4.00
χ^2^		8.37	1.14	11.60	3.39	0.01	3.06	9.82	9.38	0.55
df		1	1	1	1	1	1	1	1	1
**ε** ^2^		0.13	0.02	0.18	0.05	<0.001	0.05	0.15	0.15	0.01
*p*		<0.01	0.28	<0.001	0.07	0.94	0.08	<0.01	<0.01	0.46

Notes. N = 65; Request for ward moving: yes, *n* = 10; no, *n* = 55. Distress: 1 = never, 5 = ever; Demand, Job control, Role clarity, Relationship, and Support: 1 = ever, 5 = never; Perceived health: 1 = never, 7 = ever; Job satisfaction and Emotional workload: 1 = completely disagree, 5 = completely agree.

**Table 5:** Characteristics of the middle nurse managers interviewed.

Participant ID	Gender	Age	Years Working in Current Hospital Setting	Hospital Work Setting
ID1	F	59	over 10 years	Medical area
ID2	F	40	4 to 10 years	Critical Care Area
ID3	M	47	4 to 10 years	Services
ID4	M	60	over 10 years	Surgical Area
ID5	F	39	1 to 3 years	Surgical Area
ID6	F	42	over 10 years	Critical Care Area
ID7	F	30	4 to 10 years	Medical Area
ID8	F	54	over 10 years	Services
ID9	M	32	4 to 10 years	Operating rooms
ID10	F	55	over 10 years	Medical area

**Table 6:** Main themes and sub-themes of nurse managers’ narratives.

Themes	Subthemes	Interviewees
1. Sources of stress at work	1.1 Work overload due to too many tasks to perform	ID1, ID3, ID5, ID7, ID8, ID10
	1.2 The serious problem of staff shortages	
	1.3 Team relationships and conflict management	
2. The importance of personal and organizational support for middle nurse managers	2.1 The need for emotional support and feeling involved in the organization	ID1, ID2, ID4, ID6, ID7, ID10
	2.2 Respect for the skills of middle nurse managers and the need for support	
	2.3 Effects of personal well-being on staff	
3. Coping strategies	3.1 Personal strategies used at work	ID2, ID3, ID5, ID6, ID8, ID9
	3.2 Involvement of the work group	
	3.3 Isolation and ability to detach	
4. Actions for change to improve work activities	4.1 Motivating professionals and retaining them in the company	ID2, ID4, ID9, ID10
	4.2 Improving work tools, professional skills, and care safety	

**Table 7:** Joint display of quantitative and qualitative findings.

Quantitative Result	Corresponding Qualitative Evidence	Interview Quote	Interpretative Integration
Higher distress in middle nurse managers who requested a transfer to another unit compared with those who did not request one.	Theme 1: sources of stress at work; subtheme 1.1 workload overload and subtheme 1.2 staff shortage.	*“I am forced to constantly interrupt my work… I am never at ease” (ID7).* *“This is the most uncomfortable part of my job, because I can’t make human resources appear” (ID8).*	The request for transfer seems to be an outcome of chronic overload and organizational frustration, which the qualitative data explain as persistent stress that is not only individual but also systemic.
Lower perceived health in the group that requested a transfer.	Theme 2: importance of personal and organizational support; subtheme 2.1 need for emotional support and involvement in the organization.	*“I don’t feel supported… decisions are made directly by upper management and you just have to adapt” (ID2).* *“I would feel much more at ease if I had the opportunity to share all my concerns…” (ID10).*	Poorer perceived health seems to be linked to a sense of limited support, being less heard, and greater exposure to emotional pressure.
Lower job control in the group that requested a transfer.	Theme 2: insufficient organizational support; subtheme 2.2 tasks that do not match the role and bureaucratization of work.	*“We perform many functions that we shouldn’t be performing. We are overloaded” (ID1).* *“Each of us would need someone to help us with some of the tasks…” (ID6).*	Lower perceived control may be explained by the gap between the expected role and the role actually performed, with additional tasks and little opportunity to decide or delegate.
Lower job satisfaction in the group that requested a transfer.	Theme 2 and theme 4: lack of recognition, need for involvement, and proposals for organizational change.	*“Sometimes decisions are made directly by upper management and you just have to adapt” (ID2).* *“I would like to see an appropriate and faster IT system so that we can work better” (ID4).*	Lower satisfaction seems to be linked to limited participation and organizational barriers that reduce effectiveness, autonomy, and professional recognition.

## Data Availability

The data are not publicly available in order to protect participants’ privacy, but they may be provided upon formal request to the corresponding author.
